# Comment on: Bi-allelic variants in genes previously associated with dominant inheritance: CACNA1A, RET and SLC20A2

**DOI:** 10.1038/s41431-021-00958-y

**Published:** 2021-09-09

**Authors:** Alisdair McNeill

**Affiliations:** 1 Department of Neuroscience, The University of Sheffield, Sheffield, UK; 2 Sheffield Clinical Genetics Department, Sheffield Children’s Hospital NHS Foundation Trust, Sheffield, UK

In this issue of the *European Journal of Human Genetics*, Arteche-Lopez et al. describe novel phenotypes associated with bi-allelic variants in genes previously linked with a dominant mode of inheritance [1]. A homozygous protein-truncating variant in *CACNA1A* was identified in four infants presenting with hypotonia and epileptic encephalopathy. A number of diseases are associated with heterozygous *CACNA1A* variants: spinocerebellar ataxia with a trinucleotide expansion in the gene and episodic ataxias and migraine with single nucleotide variants. Both parents were heterozygous for the CACNA1A variants and had compatible neurological phenotypes. The severe phenotype in the children was postulated to relate to homozygous inheritance [1].

In a boy with Hirschsprung disease, a homozygous missense variant in *RET* was identified. *RET* gain of function variants is associated with multiple endocrine neoplasia, while heterozygous loss of function variants are the major risk gene for Hirschsprung disease. Neither heterozygous parent was symptomatic, perhaps because of the known reduced penetrance of *RET* loss of function variants [1].

A 55-year-old woman with primary basal ganglia calcification was identified to have a homozygous missense variant in SLC20A2. Segregation analysis suggested possible codominant inheritance, with some possibly affected heterozygotes [1].

The families reported in this paper were consanguineous; which increased the chance of bi-allelic inheritance of these autosomal dominant genes, and led to the identification of the associated bi-allelic phenotype. However, the principle that bi-allelic inheritance of autosomal dominant (i.e., a phenotype with heterozygous state) genes can be associated with a disease phenotype applies in general to clinical interpretation of genome sequencing. It also needs to be borne in mind when interpreting family histories and offering genetic counselling.

Bi-allelic inheritance of a number of autosomal dominant genes has been reported, and it is important for clinicians to be aware of these. This is increasingly recognised in rare neurodevelopmental disorders. Recently, variants in *SLC12A2* have been associated with both heterozygous and bi-allelic modes of inheritance [2]. Both heterozygous and bi-allelic variants in *SLC12A2* have been reported in association with neurodevelopmental disorders of varying severity. With homozygous variants being associated with a generally more severe phenotype. This provides further evidence of a gene dosage effect underlying genotype–phenotype correlations in such families. Interestingly, both monoallelic and bi-allelic variants in *TET3* can cause a neurodevelopmental disorder [3]. This could be explained by monoallelic variants being loss of function, while the bi-allelic variants were missense variants. The dominance or recessivity of a variant might be determined by the severity of the impact of the variant on protein stability or function. Sometimes the phenotype in heterozgyous carriers and those with bi-allelic variants can appear unrelated. For example, monoallelic missense *WDR11* variants are linked to hypogonadotrophic hypogonadism while bi-allelic loss of function variants present with a neurodevelopmental disorder [4]. It is possible that in different regions of the developing brain (e.g., hypothalamus-pituitary) a gene is haploinsufficient while in other regions (e.g., the cerebral cortex) there is no requirement for bi-allelic function of the gene.

Dual modes of inheritance are also relevant for common diseases. Bi-allelic variants in the *GBA1* gene are associated with Gaucher disease [5]. Both heterozygous carriers of *GBA1* variants and people with Gaucher disease have an increased chance of developing Parkinson’s disease. It has been postulated that this is due to reduced glucocerebrosidase enzyme activity associated with reduced lysosomal degradation of alpha-synuclein in both heterozygous and bi-allelic states. But that only severe reduction in enzymatic activity associated with bi-allelic variants leads to the more widespread clinical manifestations of Gaucher disease. Clear evidence of genotype-phenotype correlations due to gene dosage effects.

The considerations outlined above are also relevant in cancer genetics. Heterozygous variants in *neurofibromin* gene are associated with neurofibromatosis type 1, a rare neurocutaneous syndrome [6]. Inheritance of bi-allelic neurofibromin gene variants leads to a multiple primary neoplasia syndrome. This might present in paediatric oncology with unusual combinations of solid and haematological malignancies, the clinical clues to the genetic aetiology being presences of cafe-au-lait macules in the proband and possibly (sub)clinical neurofibromatosis type 1 in the parents. Ataxia telangiectasia is associated with bi-allelic *ATM* variants. However, heterozygous *ATM* variants increase breast cancer risk in carriers [7]. This should be considered when managing families affected by ataxia telangiectasia.

The research work described in this comment has clear implications for clinical practice. Clinicians should be aware of the expanding list of genes for which both dominant and recessive modes of inheritance operate. Clinicians, researchers, and bioinformaticians should consider the possibility of previously unrecognised phenotypes due to bi-allelic inheritance of dominant genes, or manifestations in heterozygous carriers of genes previously classified as autosomal recessive. The mechanisms underlying dual modes of inheritance warrant further study.
